# Advances in Electrode Design and Physiological Considerations for Retinal Implants

**DOI:** 10.3390/mi16050598

**Published:** 2025-05-21

**Authors:** Cihun-Siyong Gong

**Affiliations:** Department of Electrical Engineering, National Central University, Zhongli, Taoyuan 320317, Taiwan; alexgong@cc.ncu.edu.tw

**Keywords:** biomedical, electrode, implantable, chip, prostheses, retina, retinal, vision, visual, implant, micromachining, encapsulation, packaging

## Abstract

Until now, the ultimate solution for blind people has not been achieved, because challenges still exist. Retinal implants have emerged as a promising solution for restoring vision in individuals suffering from retinal degenerative diseases such as retinitis pigmentosa and age-related macular degeneration. Central to the efficacy of these implants is the design and functionality of the electrode arrays responsible for stimulating retinal neurons. This review evaluates the evolution of retinal implants, with particular emphasis on electrode specifications, physiological considerations for electrical stimulation, and recent advancements in electrode design. A comprehensive analysis of state-of-the-art published studies provides a detailed cross-comparison of electrode characteristics, offering insights into current state-of-the-art technologies and future directions.

## 1. Introduction

Retinal degenerative diseases, notably retinitis pigmentosa (RP) and age-related macular degeneration (AMD), lead to the progressive loss of photoreceptors, culminating in partial or complete blindness [1,2,3,4,5,6,7,8,9,10]. RP is a rare inherited condition (affecting approximately 1 in 4000 people worldwide) and a leading cause of inherited blindness, while AMD is a common cause of vision loss in older adults, affecting tens of millions globally. These conditions spare the inner retinal neurons initially, but the absence of photoreceptor input renders the visual pathway non-functional. Other less prevalent degenerative diseases (e.g., choroideremia or cone–rod dystrophies) that result in photoreceptor loss may also be potential targets for retinal implants. Retinal implants (also known as retinal prostheses or retinal “chips”) aim to bypass damaged photoreceptors by electrically stimulating the remaining viable retinal neurons, thereby restoring partial vision [11,12,13,14,15,16,17,18,19,20]. The success of these devices hinges on the precise design and implementation of electrode arrays that interface seamlessly with retinal tissue. These implants can be mainly categorized into two popular types, subretinal and epiretinal, each with distinct mechanisms and design considerations. This review presents a detailed comparison of subretinal and epiretinal implants, examining their structural differences, electrode configurations, electrical stimulation strategies, and clinical outcomes. Furthermore, the limitations and future directions for each type are discussed to guide ongoing research and development. The technical relationship between retinal implants and integrated sensing and transducing devices (ISTD) lies in their shared reliance on advanced microelectronics and sensor technologies to interface with biological systems. The front end of an ISTD is critical to the success, as it interfaces directly with the retinal tissue. As a result, recent advances in retinal electrode design, including their challenges, are also discussed in this paper. Compared to prior reviews of retinal prosthetics that covered earlier developments, this work emphasizes up-to-date technological developments (through 2024) and provides a unique focus on how electrode engineering choices influence physiological outcomes and long-term clinical performance.

## 2. Survey of Retinal Implants and Their Stimulation Strategies

**Figure 1:**
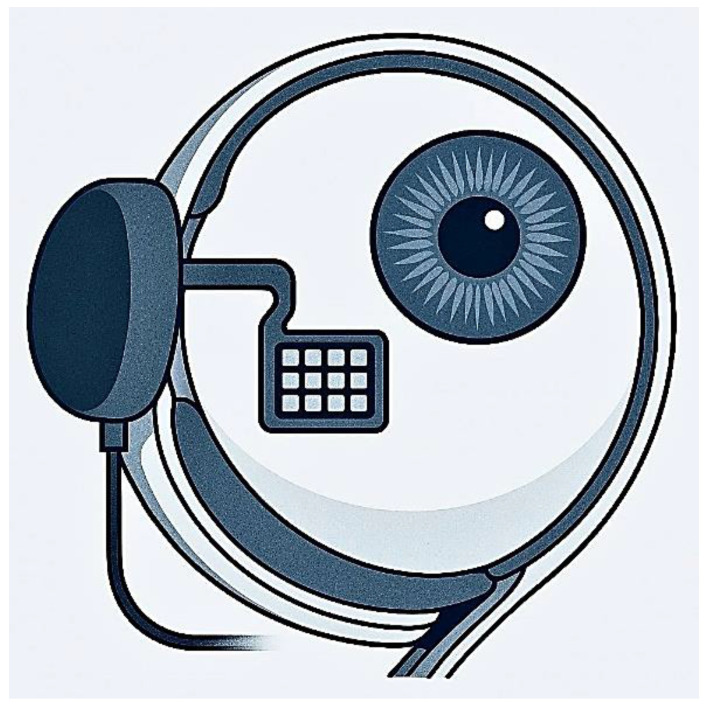
Implantable retinal devices for blind individuals.

Retinal chips or implants, such as those developed for retinal prostheses (e.g., the Argus II or similar systems), are designed to restore vision in individuals with degenerative retinal diseases like RP or AMD. Specifically, they replace the function of degenerated photoreceptors by electrically stimulating the remaining retinal neurons. Figure 1 shows a conceptual drawing of a retinal prosthetic system. These devices typically consist of an array of microelectrodes implanted onto or near the retina. They work by converting visual input (often captured by an external camera) into electrical signals that stimulate surviving retinal cells, bypassing damaged photoreceptors. The stimulated cells then transmit signals via the optic nerve to the brain, which interprets them as visual perceptions. The classification of retinal implants depends on their placement within the eye.

Structurally speaking, epiretinal implants are mounted on the retinal surface and deliver electrical impulses directly to ganglion cells and their axons [21,22,23,24,25,26,27,28]. These devices are usually coupled with an external camera and image processor, which transmits visual data wirelessly to the implant. With regard to their electrode configurations, planar (surface) electrode arrays provide broad activation fields, whereas three-dimensional (3D) microelectrodes protruding into tissue can improve contact efficiency and selective stimulation. High-density arrays are also being explored to enhance visual acuity and contrast sensitivity. In terms of signal processing, temporal encoding schemes convert visual scenes into pulsatile stimulation patterns, and current steering techniques help improve spatial selectivity and reduce unwanted crosstalk between electrodes. Devices like the Argus II system exemplify the epiretinal approach: the implant is positioned on the retina surface to directly stimulate ganglion cells, with an external camera capturing visual information that is then processed and transmitted wirelessly to the implanted electrode array [29,30,31].

By contrast, subretinal implants are inserted beneath the retina (between the neural retina and the retinal pigment epithelium, RPE), replacing the function of lost photoreceptors by stimulating the remaining bipolar cells from below. These devices leverage residual retinal circuitry for signal processing. Subretinal implants often integrate microphotodiodes that convert incident light into electrical current, mimicking the natural phototransduction process within the eye. The PRIMA system by Pixium Vision is a notable example, employing a 2 mm square chip implanted under the retina to restore vision in patients with atrophic AMD [32,33,34,35,36]. Its design includes thin-film microelectrode arrays made from biocompatible materials (e.g., platinum and iridium oxide), high-density electrode layouts to improve spatial resolution, and flexible substrates to conform to the curved retina and reduce mechanical stress on the tissue.

**Figure 2:**
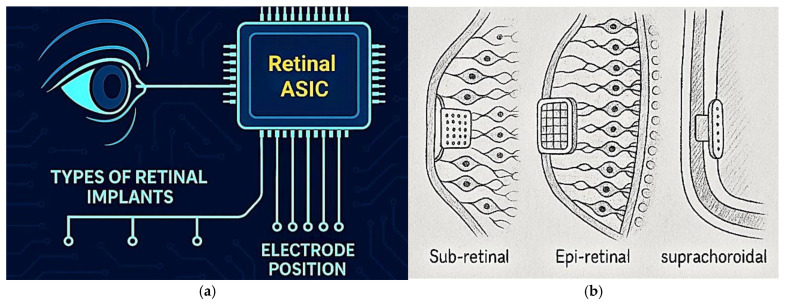
General device functionality (**a**) and electrode placement (**b**) for retinal prostheses.

Figure 2 shows both the overall system and implant placement for these approaches. Figure 2a depicts the general functionality of a retinal prosthesis system (camera, processor, transmitter, and retinal implant), and Figure 2b illustrates the difference in electrode placement for epiretinal vs. subretinal implants. Epiretinal arrays sit on the inner retinal surface, whereas subretinal arrays are situated beneath the retina in the subretinal space.

There are also suprachoroidal implants, placed in the suprachoroidal space (between the choroid and sclera) to stimulate retinal neurons, although they are not traditionally categorized as “retinal” implants due to their more distant positioning. Like their epiretinal counterparts, suprachoroidal implants use an electrode array (often paired with an external camera) to generate phosphenes (light percepts). Advantages of suprachoroidal placement include safer, less invasive surgery, and implant stability (with electrodes secured in the scleral pocket) as well as preservation of any residual natural vision. However, disadvantages include the need for higher stimulation currents (due to the greater distance from target neurons, leading to lower spatial resolution) and surgical risks like choroidal bleeding. Recent developments in this area focus on higher-channel-count devices (e.g., the 44-channel Phoenix99 suprachoroidal implant) and improved electrode designs, with early trials showing that suprachoroidal prostheses can elicit useful visual percepts with a favorable safety profile. Table 1 compares the key characteristics of the two major implant types, epiretinal and subretinal. As shown, each approach presents unique advantages and challenges.

With regard to the stimulation strategies employed, two primary types of electrical stimulation paradigms have been implemented in retinal implants: (1) photovoltaic-based stimulation, in which incident light is converted into electrical current by on-chip photodiodes (thereby eliminating the need for an external power source), and (2) current-controlled stimulation via an implanted receiver that provides power and data to the electrode array (allowing precise control of current pulses delivered to tissue). Both approaches require optimization of pulse frequency and duration to ensure effective activation of retinal cells, while minimizing adaptation or damage. Each approach has distinct advantages and challenges concerning power supply, signal processing, and surgical complexity, as noted above. Regardless of approach, neural adaptation mechanisms (such as dynamic gain control or feedback from the patient’s perceptual response) can be used to fine-tune stimulation parameters over time. Retinal implants, to date, have demonstrated significant potential, but challenges remain in fully optimizing their performance and patient outcomes. In view of these challenges, ongoing developments in the field are focusing on several key factors:

1. Enhanced electrode designs via nanotechnology—for improved biocompatibility and stimulation efficiency (e.g., nano-structured electrode surfaces to increase charge injection capacity).
2. Hybrid implant systems—combining aspects of both epiretinal and subretinal approaches to maximize benefits (for instance, devices that have both subretinal photodiodes and epiretinal stimulation electrodes).
3. Artificial intelligence (AI) integration—leveraging AI for real-time adaptation of stimulation patterns and image processing.
4. Personalized stimulation protocols—tailoring electrode activation based on individual neural responses (using patient-specific models or feedback to adjust stimulation).
5. Minimally invasive surgical techniques—to reduce risks and improve long-term device stability (such as novel implantation tools and procedures to place implants with less trauma).

These focal areas aim to address current shortcomings and usher in the next generation of retinal prosthetic technology.

## 3. ISTD Concepts Applied to Retinal Implants and Current State-of-the-Art

Integrated sensing and transducing devices (ISTDs) align closely with the core functionality of retinal implants. A retinal prosthesis effectively integrates sensing (capturing visual information via a camera or photodiodes) and transducing (converting that information into electrical stimuli for neurons) within a single system—embodying the ISTD principle in a vision restoration context. The technical overlap between retinal implants and other ISTDs includes:

- Microfabrication and miniaturization: Both rely on microelectromechanical systems (MEMS) and advanced fabrication techniques to create compact and biocompatible devices. For example, retinal chips use microelectrode arrays made of materials like silicon, platinum, or polyimide, that are similarly employed in other neural ISTDs for precision and durability.
- Signal processing: In retinal implants, as external camera or implanted photodiode array captures visual data, which are then processed by microelectronics and transduced into patterned electrical pulses. This mirrors ISTD principles, wherein sensing and actuation are tightly coupled. The implant’s ability to encode visual information into neural-friendly signals is a prime example of an ISTD in action.
- Biointerface: Both retinal implants and other neuroprosthetic ISTDs require a seamless interface with biological tissue. Retinal implants must stimulate neurons effectively without causing chronic damage, a challenge shared by devices like cochlear or cortical implants. This necessitates optimizing electrode materials, impedance, and stimulus waveforms, which are common engineering considerations across ISTDs.
- Wireless power and data transmission: Modern retinal implants often use wireless power (e.g., inductive coupling) and telemetry to eliminate transcutaneous wires, a feature also seen in advanced ISTDs. This enables continuous operation without tethering the patient, but requires careful design to maintain reliability and safety.

**Figure 3:**
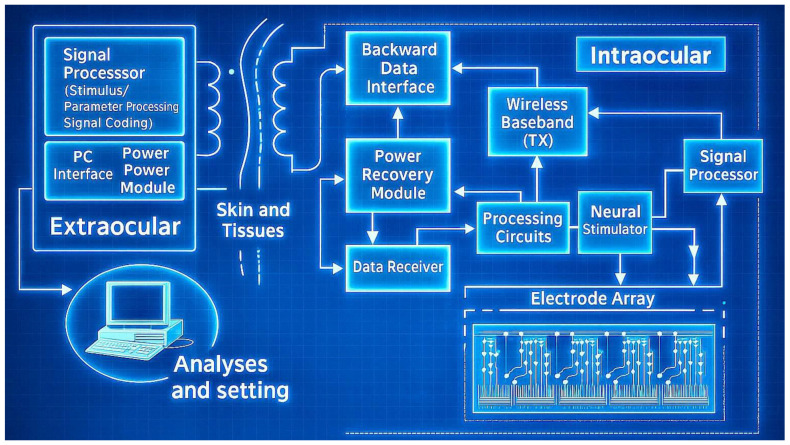
A wireless implantable system architecture for retinal electronic devices [37].

Figure 3 illustrates a typical wireless retinal prosthesis architecture. It consists of an external unit (camera and processor) that sends power and data wirelessly to an internal receiver coil, which then drives the microelectrode array in the eye [37]. Such designs demonstrate the ISTD concept by integrating sensing (image capture) and transducing (neural stimulation) components. Projects like the Argus II epiretinal prosthesis and the Alpha-AMS subretinal chip (Retina Implant AG) exemplify these ISTD principles in practice [38,39,40,41]. A comparative overview of major retinal implant systems is provided in Table 2 [38,39,40,41,42,43,44,45,46,47]. These devices integrate light sensing (via camera or photodiodes) and stimulation on a chip to create artificial vision, essentially functioning as specialized ISTDs for the visual system. Effective electrical stimulation of the retina necessitates a nuanced understanding of retinal physiology, the electrical properties of neural tissue, circuit design constraints, and electrode fabrication—i.e., multiple interdisciplinary considerations that are similarly crucial in any ISTD. Micromachining technology (such as 3D printing, deep reactive ion etching (DRIE), and laser ablation) enables the creation of microscale structures with high precision, which is essential for developing dense and effective electrode arrays. Key design considerations include:

1. Electrode–retina distance: Minimizing the gap between electrodes and target neurons lowers the required stimulation threshold and enhances spatial resolution.
2. Electrode size and geometry: Smaller electrodes offer higher spatial selectivity but may require higher current densities (raising risk of tissue damage), whereas larger electrodes deliver current over a broader area with lower density but reduce specificity.
3. Charge density and pulse parameters: Safe stimulation mandates adhering to charge-injection limits to prevent electrode corrosion or tissue damage. Optimizing the pulse waveform (duration, amplitude, and frequency) is crucial for effective yet safe neural activation.
4. Material biocompatibility: Electrodes must be made of materials that are biocompatible and capable of consistent electrical stimulation over years. Traditional materials like platinum and iridium oxide are the gold standards, and emerging alternatives like graphene are under investigation for their flexibility and conductivity.

Recent advancements in retinal prostheses have increasingly focused on enhancing visual outcomes and long-term patient satisfaction. One direction has involved increasing electrode count and density to improve visual acuity. For instance, the upcoming NR600 epiretinal prosthesis (by Nano Retina) features an array of hundreds of microelectrodes with proprietary circuitry for highly localized stimulation, aiming to significantly improve spatial resolution compared to earlier devices [48,49,50,51,52,53]. Another direction is fully wireless, photovoltaic implants (like Pixium’s PRIMA), which eliminate external power tethers by converting light (from special glasses that project infrared images) into electrical stimulation on the chip [54,55,56,57]. These designs emphasize biocompatibility and efficient energy conversion to safely stimulate retinal neurons. Notably, photovoltaic systems must operate within ocular safety limits for light intensity to avoid heating tissue—an important engineering constraint. Researchers have also begun incorporating computational models and AI algorithms to optimize electrode configurations and stimulation patterns. For example, greedy optimization algorithms and conditional invertible neural networks have been explored in simulation studies to optimize which electrodes to activate and how, in order to maximize the quality of the perceived image [58,59,60,61,62,63]. These intelligent approaches can potentially adjust stimulation in real-time to account for variability in retinal response, although they introduce additional computational complexity.

Table 2 provides a historical overview and comparison of several landmark retinal implant systems, highlighting their main contributions, advantages, and limitations, as well as whether they involved epiretinal or subretinal devices.

## 4. Advances in Electrode Design

Research suggests that recent advances in retinal electrode design focus on flexibility and biocompatibility. Novel materials and structures are being employed to ensure that electrodes conform to the tissue and remain stable over time. For example, engineers have begun using materials like liquid metals (e.g., gallium–indium eutectic alloys) and liquid crystal polymers (LCP) as substrates [64,65,66,67]. These materials offer a combination of softness and durability: liquid metals can deform without breaking, and LCP is a biocompatible polymer with very low moisture absorption. It is likely that advanced micromachining techniques—such as 3D printing and DRIE (deep reactive ion etching)—will be crucial for creating precise, high-density electrode arrays from these materials.

**Figure 4:**
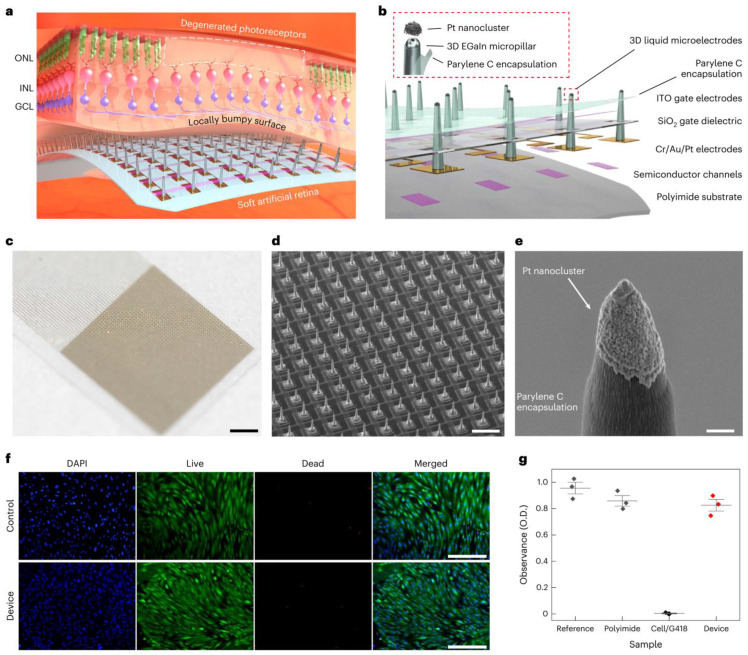
Flexible artificial retina featuring 3D liquid metal (LM) microelectrode arrays [79]. (**a**) Diagram illustrating the artificial retina equipped with 3D LM microelectrodes positioned near the uneven retinal surface. The outer nuclear layer (ONL), inner nuclear layer (INL), and ganglion cell layer (GCL) are labeled accordingly. (**b**) Diagram showing the structure of the artificial retina, combining light-sensitive transistors with 3D LM microelectrodes. (**c**) Image of the artificial retina, showcasing a high-resolution transistor array paired with 3D LM microelectrodes. Scale bar: 1 mm. (**d**) Scanning electron microscopy (SEM) image displaying the high-resolution phototransistor array (50 × 50 pixels; pixel spacing, 100 μm) integrated with 3D LM microelectrodes, each 60 μm tall, prior to the application of the upper Parylene C protective coating. (**e**) SEM image revealing platinum nanoclusters (PtB) selectively applied to the tip of the 3D LM stimulation electrode. Scale bar: 1 μm. This procedure was independently performed over ten times, producing similar results each time. (**f**) Typical fluorescence microscopy images showing DAPI and live/dead staining of human retinal cells grown on the device with 3D LM microelectrodes. Scale bars: 400 μm. This test was independently repeated three times with comparable results. (**g**) Cell viability assessment of the artificial retina. O.D. refers to optical density. Data represent the mean ± standard deviation (s.d.), based on three independent experiments (n = 3) [75].

**Figure 5:**
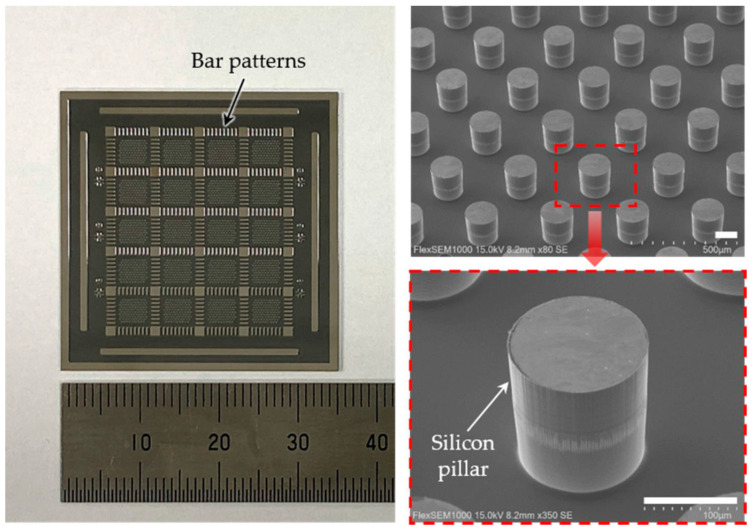
(**Left**) Image of a chip featuring 20 arrays of circular silicon pillars, measuring 35 mm by 35 mm. (**Right**) Scanning electron microscopy (SEM) images showcasing silicon pillars following deep reactive ion etching. The scale bars represent 100 μm [76].

Micromachining technologies enable the fabrication of tiny, complex electrode structures with exact shapes and sizes, ensuring they fit well against the curved retina and function effectively. Techniques like DRIE and laser machining help to create electrodes with exact shapes and sizes, ensuring they fit well and work effectively [68,69,70,71]. This capability supports the development of high-resolution devices that could significantly improve patients’ vision, with some designs theoretically approaching visual acuities on the order of 20/160 under optimal conditions. Of course, challenges like long-term stability and implantation safety remain to be addressed, even as resolution improves [72,73]. The evolution of electrode design in retinal implants has been pivotal in improving device performance and patient outcomes. Discussed below are some of the most recent exciting advances in the field of electrodes associated with these applications. Some key trends and breakthroughs include:

1. Use of Novel Materials: The exploration of novel materials, such as graphene, has led to the development of electrodes that are not only both flexible and biocompatible, but also reduce the risks of mechanical damage to the retina and chronic immune responses. For instance, the incorporation of graphene (an ultra-thin, flexible carbon sheet) has led to electrodes that conform to tissue while maintaining excellent conductivity. Similarly, carbon nanotube (CNT) microelectrodes have shown promise—their high surface area and conductivity allow lower stimulation thresholds and intimate integration with neural tissue [74]. A cutting-edge development, detailed in a 2024 study, involves the use of soft, biocompatible liquid metals (LMs) such as eutectic gallium–indium alloy (EGaIn) for retinal electrodes [75]. As shown in Figure 4, these electrodes are integrated into an ultrathin (10-μm) artificial retina, combining flexible phototransistors with 3D micropillar electrodes. The electrodes are 3D micropillar arrays, with a height of 60 μm and diameter of 20 μm, scalable down to 5 μm. They are coated with platinum nanoclusters at the tips to enhance charge injection, achieving a charge storage capacity of 72.84 mC cm^−2^ and impedance of ~210 kΩ at 1 kHz. In vitro tests showed 82% cell viability, and in vivo tests in animal models revealed no inflammation or damage after 5 weeks, indicating suitability for long-term implantation. The major advantage of this development is the flexibility in minimizing the electrode–cell distance, reducing activation thresholds and improving spatial resolution. This design supports less invasive epiretinal implantation, potentially achieving 20/160 vision with high-resolution devices, a significant step toward functional vision restoration.
2. Three-Dimensional Electrode Arrays: Advancements in microfabrication have enabled creation of 3D electrode geometries that better match the curvature and layered structure of the retina. Arrays of microscopic pillars or needles can penetrate or closely approach retinal neurons, ensuring consistent contact and effective stimulation. These 3D designs help minimize the electrode–retina distance (which is critical for lower thresholds and higher resolution). For example, a 2020 study introduced a subretinal 3D microelectrode array arranged in a hexagonal pattern to improve focal stimulation, as shown in Figure 5 [76]. This device used 98 pillar electrodes (150 μm diameter, ~20 μm high) on a flexible PDMS base, achieving a mean impedance of ~385 kΩ at 1 kHz and a charge storage capacity (CSC) of 2.83 mC·cm^−2^. This design aims to enhance visual acuity by reducing current dissipation and improving the activation of retinal cells. Such structured arrays demonstrated enhanced localization of stimulation and improved retinal cell activation compared to planar electrodes. A 2013 study highlights the use of liquid crystal polymer (LCP) for retinal electrodes, noted for its low moisture absorption compared to polyimide, Parylene, and silicone elastomers [77]. LCP is used in a novel retinal prosthetic device with monolithic encapsulation, integrating neural stimulation circuitries into a thin, eye-conformable structure. The study details electroplating for thickened metal tracks to improve mechanical strength and long-term reliability, enabled by high-pressure lamination. Laser machining, including laser ablation and laser thinning, enhances flexibility, particularly for the LCP electrode. A 2019 review also mentions the emerging field of tissue electronics using organic conductive/semi-conductive polymers, which offer more physiological interaction and potentially higher spatial resolution, fabricated using micromachining for precise patterning in terms of electrodes [78].
3. Current Steering and Multiplexing: Implementing current steering strategies allows modulation of electric fields to stimulate target neurons selectively, without needing a one-to-one correspondence between electrodes and perceptual pixels. By dynamically controlling the distribution of current between neighboring electrodes, researchers can create “virtual” electrodes that effectively increase resolution. This can enhance spatial resolution without physically increasing the electrode count, mitigating some design and fabrication challenges. Current steering, along with multiplexing (time-sharing of electrodes), also helps manage power consumption and heat dissipation, which is crucial for devices with tens to hundreds of channels.
4. Prosthetic Vision Simulation and Optimization: There is growing use of computational models and simulations of prosthetic vision to guide electrode layout. By simulating how a patient perceives patterns of stimulation, researchers can identify electrode configurations that maximize the visual field coverage or improve the clarity of perceived patterns. For example, recent studies employed algorithms (including AI-based approaches) to optimize electrode placement for better visual outcomes. These simulations inform design decisions, such as where to place higher density clusters of electrodes or how to arrange them to avoid interference, ultimately influencing hardware prototypes.

To illustrate some state-of-the-art electrode designs, Table 3 summarizes a selection of recent advances (from ~2013 to 2024). This includes both experimental research devices and notable engineering demonstrations that push the boundaries of electrode performance. As seen above, each new design brings certain advantages (e.g., higher charge injection, flexibility, or better targeting of neurons) while addressing specific limitations of previous designs. It is important to note that many of these advances are at the preclinical or prototype stage. Translating them into clinical devices will require ensuring their long-term reliability and safety. These topics are addressed in the next section.

## 5. Comparative Properties of Electrode Materials

The choice of electrode materials can greatly influence the performance and biocompatibility of a retinal implant. Key factors include the material’s electrochemical stability (can it endure repeated pulsing without corrosion?), its biocompatibility (does it cause inflammation or toxicity?), and its mechanical properties (is it flexible or rigid, and how does that affect the tissue interface?). Table 4 compares several common and emerging electrode materials on these points. This table provides specific examples of metals, metal oxides, carbon nanomaterials, and polymers used in retinal prostheses.

## 6. Encapsulation and Packaging Technology (Biocompatibility Considerations)

Implantable medical devices play a critical role in clinical applications, with their core component being highly integrated chips. However, these chips must operate reliably over extended periods within the harsh environment of the human body. Consequently, effective encapsulation/packaging to ensure functional stability, biocompatibility, and long-term reliability has emerged as a pivotal technological challenge [80,81]. The development of encapsulation/packaging techniques must address multiple requirements, including miniaturization, biocompatibility, moisture and gas barrier properties, electrical connectivity, thermal stability, and mechanical integrity. This section reviews encapsulation materials, architectures, and key technologies for implantable retinal devices, offering a comparative analysis of their advantages and disadvantages. Recent trends aimed at improving encapsulation performance are also highlighted.

Encapsulation/packaging materials for implantable devices must exhibit excellent biocompatibility to prevent immune responses or toxicity when interacting with human tissues. Additionally, these materials require resistance to corrosion and hydrolysis to ensure the chip’s long-term stability in vivo [82]. Moisture infiltration can lead to internal short circuits and corrosion, damaging circuits and chips. Thus, encapsulation structures must achieve an extremely low water vapor transmission rate (WVTR), typically below 10^−6^ g/day·cm^2^ [79]. Additionally, the encapsulation must maintain stable electrical connections and provide adequate heat dissipation to prevent tissue heating during device operation [83]. Common encapsulation materials and approaches include:

1. Polyimide (PI): Polyimide offers superior thermal stability and mechanical strength, making it popular for flexible electronics and ribbon cables in implants [69,84]. It is moderately moisture-resistant but can still allow slow ingress; thus thin-film metal or ceramic coatings may be added for long-term hermeticity.
2. Parylene C: This is a vacuum-deposited polymer coating that forms an ultra-thin conformal layer. Parylene-C has excellent biocompatibility and low permeability, making it useful as a coating on electronics. Its drawbacks are limited mechanical strength and potential cracking under flexing, but it is often used in combination with other encapsulation (e.g., a Parylene-coated device further encased in silicone) [85,86].
3. Silicone (PDMS): Polydimethylsiloxane (PDMS) is widely used because of its flexibility, biocompatibility, and ease of use. However, silicones are relatively permeable to gases and fluids, so they often need additional barrier layers (like inorganic coatings) to prevent water ingress [84,87].
4. Ceramics and Metals: Traditional hermetic packages (like those in pacemakers) use welded titanium cases or ceramic enclosures (e.g., alumina). These provide superb moisture barrier properties and long-term stability [82]. High-density ceramics such as alumina (Al_2_O_3_) and aluminum nitride (AlN) provide exceptional gas barrier properties and heat resistance, commonly used in long-term, high-reliability implantable devices [88]. Titanium, with its outstanding biocompatibility and mechanical strength, is a preferred material for traditional pacemaker and neurostimulator casings. However, its opacity and rigidity limit its use in applications requiring optical sensing or flexibility [88].
5. Multilayer Encapsulation and Others: To overcome the limitations of single-material systems, multilayer encapsulation architectures—such as alternating silicon oxide/silicon nitride stacks or composites of Parylene with inorganic materials—are increasingly adopted, offering improved moisture resistance and mechanical protection [89]. Moreover, many modern implantables impose higher demands on the electromagnetic transparency and design flexibility of encapsulation materials [90]. MEMS and integrated encapsulation/packaging designs enable high-density integration and miniaturization through wafer-level packaging (WLP) and thin-film encapsulation techniques [91]. Laser bonding offers a high-precision, low-temperature process suitable for ceramic or glass encapsulation systems with stringent gas barrier requirements, commonly applied in retinal implants and high-density neural interfaces [92]. To systematically compare encapsulation approaches, Table 5 summarizes several encapsulation types and their advantages and disadvantages.

In addition to materials, packaging architectures vary. Some implants use a fully encapsulated bulk electronics module connected via a sealed cable to a separate electrode array (e.g., Alpha-IMS has an electronics capsule with a ribbon cable to the subretinal chip). Others, like PRIMA, have minimized packaging by simplifying electronics (photodiodes on chip require no internal power source, so they can be encapsulated by only thin dielectric layers). Each architecture has trade-offs in complexity, profile, and reliability. With advancements in system-on-chip (SoC) design, the ongoing miniaturization and even nanoscale reduction of chip sizes necessitate encapsulation that supports smaller volumes and greater functional integration. Co-design of chips and encapsulation is poised to become mainstream [93]. A key research focus is accelerating the simulation of decades-long encapsulation aging effects within the body, establishing effective accelerated life testing models to validate reliability [94]. In the realm of degradable materials and temporary implants—particularly for short-term diagnostic or therapeutic applications—biodegradable encapsulation is an emerging field. Such systems aim to degrade naturally and be absorbed by the body after fulfilling their purpose, reducing surgical risks [95]. The following is a concise introduction and analysis of selected significant studies from the past 25 years:

Cui et al. utilized conductive polymers (e.g., PEDOT:PSS) to modify neural electrode surfaces, enhancing conductivity and providing a favorable environment for cell adhesion, thus improving long-term signal stability. However, the long-term stability and thermal resilience of these materials remain limited, and their performance can be affected by protein adsorption in bodily fluids [95]. Rickert et al. developed flexible microelectrode arrays that conform to brain curvature, reducing micro-damage from mechanical stress. This technology offers high signal quality and extended lifespan, although its complex multilayer structure poses challenges in process control and reliability validation [96]. Ghaffari et al. created a sweat sensor integrating microfluidics and encapsulation, enabling simultaneous encapsulation, fluid transport, and chemical sensing. However, sealing and long-term stability of the fluid channels require further improvement [97]. Researchers have developed biodegradable encapsulation materials such as polylactic acid and bio-silicon, which naturally degrade after treatment, eliminating the need for removal surgery. However, material options are limited, and controlling the encapsulation lifespan remains challenging, rendering them unsuitable for long-term implants [98,99,100]. Iqbal proposed design guidelines for wireless energy transfer and data integration in encapsulation, offering recommendations on antenna placement, encapsulation thickness, and material electromagnetic properties [101]. While critical for enhancing device performance, trade-offs between penetration depth and efficiency persist [102].

Barrese et al. systematically analyzed failure mechanisms in implantable electrodes, identifying material cracking, micro-crack infiltration, and encapsulation interface delamination as primary issues, providing a basis for subsequent improvements [103]. Fallegger et al. developed soft encapsulation for neural interfaces with integrated wireless transmission and power supply, balancing thinness and hermeticity. However, stability under dynamic biological conditions requires further enhancement [104]. Hossain et al. highlighted that MEMS encapsulation integrates sensing, signal processing, and transmission components into a single system, achieving miniaturization and high functionality. Challenges remain in thermal management and process consistency [105]. Ahn et al. systematically reviewed encapsulation technologies and their evolution, identifying trends such as “hybrid multi-material encapsulation”, “smart encapsulation”, and “miniaturized high-integration design”. The trade-off between hermeticity and flexibility remains a core focus for future development [106].

Last but not least, biocompatibility is crucial to the clinical success of retinal wafers. Five recent retinal wafer biocompatibility research results were selected from the literature for a simplified analysis and comparison as follows: Jurak et al. reported on the recent progress of biocompatible materials, emphasizing the low immunoreactivity of polymers and nanomaterials, but pointed out that long-term stability still needs to be improved for the preliminary design of retinal implants [107]. Eggenberger et al. tested the long-term biocompatibility of suprachoroidal implants in a sheep animal model and found good tissue integration and low inflammatory response, but electrode material degradation issues affected long-term function [108]. Spicer et al. proposed optimizing materials (such as biocompatible nanocomposite material) to acoustically stimulate cells in the diseased retina, showing promise in sending signals to the brain’s visual cortex [109]. Nguyen et al. investigated in vivo biocompatibility study for novel graphene electrode applied to the retinal implants, and their results showed that this new carbon-based material with a large water window is able to provide exceptional electrical performances for neurophysiological recording and capable charge injection for neuronal stimulation [110].

In summary, the encapsulation and packaging of retinal prostheses is a critical aspect that underpins long-term success. Looking forward, encapsulation technology is advancing toward: 1. High-density feedthroughs that can support hundreds of electrode channels without compromising hermeticity. 2. Flexible encapsulation methods (e.g., thin-film multi-layer coatings or LCP encapsulation) that combine hermetic sealing with device flexibility. 3. Integrative designs where the packaging is not an afterthought but built into the device fabrication (as in monolithic LCP devices or fully polymer-based implants). 4. Active monitoring—some designs even utilize built-in sensors to detect moisture ingress or stress, allowing early intervention or shutdown of the device for safety. Table 6 summarizes some notable examples from the literature of implantable device packaging techniques, including applications beyond the retina (since many lessons learned from brain or cardiac implants can translate to retinal devices).

## 7. Conclusions and Future Directions

Retinal implants—also referred to as retinal prostheses or retinal “chips”—have been a major focus of neuroengineering research over the past 25 years, aimed at restoring vision to individuals with retinal degenerative diseases. In this review, we have surveyed a broad range of scientific studies published between 2000 and 2025, with emphasis on retinal prosthesis technologies, electrode innovations, and clinical trial outcomes. The landscape of retinal implants has evolved significantly, particularly in electrode design and stimulation strategies. Subretinal and epiretinal implants each offer distinct advantages and limitations. Subretinal implants, by virtue of their placement, can leverage intrinsic retinal processing (stimulating bipolar cells upstream in the visual pathway) and often feature a higher electrode density, contributing to potentially better visual acuity outcomes. Epiretinal implants, on the other hand, offer easier surgical implantation and the flexibility of external hardware adjustments (since they rely on an external camera and processor). This dichotomy is evidenced in clinical trials: for example, the subretinal Alpha-IMS/AMS enabled some patients to read large letters and discern objects at improved resolution compared to earlier epiretinal devices, whereas the epiretinal Argus II—while easier to implant—provided more limited acuity (around 20/1260 in the best cases) but still significantly improved patients’ orientation and mobility in daily life. Encouragingly, advances in electrode materials, stimulation paradigms, and computational models are steadily enhancing the performance of these implants. Recent electrode designs aim for increased flexibility to reduce tissue damage (for instance, using soft liquid metals or polymers that conform to the retina) and higher electrode counts for finer vision.

Micromachining techniques like 3D printing have enabled complex 3D electrode architectures that improve stimulation focus. Unexpectedly, artificial intelligence algorithms have also begun to play a role: for example, researchers have used machine learning (e.g., conditional neural networks and greedy optimization) to optimize stimulus patterns and electrode configurations. These approaches can personalize and adapt stimulation in ways that were not previously possible. While AI-driven strategies are still in the experimental stages, initial results are promising, suggesting that future retinal implants might automatically adjust to each user’s neural responses or even to the content of the visual scene. Potential challenges, such as increased computational load and the need for real-time performance, must be considered when introducing AI control. Despite detailing many technical advantages throughout this review, it is equally important to consider the limitations of current retinal prosthetic technology. To date, even the most advanced implants restore only partial vision—often just spots of light or crude shapes. Many Argus II users, for instance, perceive a pixelated array of flashes and require substantial training to interpret visual patterns. Typical visual acuity with these devices remains in the range of hand motion to counting fingers (far from the 20/20 of normal vision). Field of view is another issue: most devices provide a limited visual field (e.g., ~20° for Argus II, or a small central patch for PRIMA), which means users must scan actively to form a complete image of their surroundings.

There are also patient-to-patient variations—some individuals achieve better functional outcomes than others, due to factors like residual retinal health, optic nerve status, and even how the brain adapts to the artificial input. Psychologically, while many recipients are grateful for restored light perception, they also note the artificial nature of the vision (monochromatic phosphenes, lag from the camera in epiretinal systems, etc.). Managing expectations and providing rehabilitation is crucial in the clinical adoption of these devices. From a technical perspective, key limitations include long-term stability (as discussed, electrode encapsulation and tissue responses can lead to performance degradation over years) and power/data constraints. Wireless power delivery must avoid tissue heating—a real concern, since inductive coupling or infrared illumination can cause local temperature rises. Modern devices mitigate this by efficient design and pulsed operation, but the total energy that can be safely delivered is limited.

This in turn limits the number of electrodes that can be driven simultaneously and the strength of stimuli, potentially limiting visual function. Future implants need to improve power efficiency or use alternative strategies (e.g., on-chip energy storage or more sensitive photovoltaic materials) to overcome this barrier. Another concern is safety and failure modes: a failure in a chronic implant could potentially harm the patient (for example, an electrical short could damage tissue or a breached package could cause inflammation). Fortunately, clinical trials so far indicate that serious adverse events are rare—for instance, the Argus II 5-year follow-ups reported no device-related blindness progression, aside from the expected course of disease, and most issues were addressed by device replacements or repairs. Nevertheless, ensuring regulatory approval will require demonstrating robust safety margins (e.g., that no single-point failure can cause an unsafe output). This is an area where improvements in packaging, self-monitoring of devices, and failsafe circuit design will be beneficial. Looking ahead, the field is moving toward wider clinical adoption of the most promising technologies. Two notable examples are the aforementioned PRIMA (Pixium Vision) and NR600 (Nano Retina) systems. PRIMA is a wireless subretinal microchip; a recent 4-year clinical study in AMD patients showed that it enabled some letter reading and had maintained function with no major safety issues.

The NR600, an intraretinal (partially penetrating) device, has completed an initial multi-center trial demonstrating its basic safety and an ability to elicit structured visual perceptions in RP patients. These results are encouraging, but before such devices can become mainstream, several challenges must be addressed. Regulatory and cost barriers are significant: the commercial discontinuation of Second Sight’s Argus II in 2019 (despite FDA approval) underscored that high device cost and modest efficacy can limit market viability. Future implants will need not only to perform better, but to do so cost-effectively to gain support from healthcare systems. In this context, simplifying designs (to reduce manufacturing costs) or improving outcomes (to justify higher costs) are two viable paths. From a patient perspective, the quality of vision remains the ultimate metric. Feedback from recipients suggests that even limited vision can greatly improve independence—patients report enjoyment in detecting light, locating high-contrast objects, or seeing motion that was previously absent. However, they also express a desire for improvements such as color vision, higher resolution (to perhaps recognize faces or read unconstrained text), and a broader visual field. These user-driven goals align with current research directions: efforts in biomaterials, electronics, and computational imaging all converge on creating a prosthesis that feels more natural and yields more meaningful visual information. In short, retinal prosthetics have progressed from conceptual experiments to bona fide clinical devices assisting blind individuals, but there is still a long road ahead to reach widespread adoption. The next generation of retinal implants will likely incorporate:

1. Higher-density electrodes (possibly leveraging nanoscale features or 3D architectures to approach visual acuity in the ranges of legal blindness or better, e.g., 20/200 to 20/100);
2. Advanced image processing (potentially AI-driven algorithms for feature enhancement, edge detection, and real-time adaptation to the environment and patient feedback);
3. Improved materials (such as fully organic or hybrid materials that reduce foreign body response and last the lifetime of the patient without replacement);
4. Modular or upgradable systems (for example, external components that can be improved without replacing the implant, as seen with some upgradeable vision prosthesis platforms).

Interdisciplinary collaboration between ophthalmologists, neuroscientists, and engineers will continue to be essential. Each improvement in hardware must be matched with understanding of how the visual system responds. The ultimate goal remains restoring meaningful vision—not just flashes of light, but functional vision that can aid in daily activities. While achieving normal sight is not yet within reach, the developments reviewed in this paper make it clear that, step by step, the gap is closing. Retinal implants are steadily moving from the realm of experimental trials toward becoming a life-changing option for those with incurable blindness. With continued research and carefully guided clinical studies, it is hoped that these “bionic eyes” will eventually allow blind individuals to navigate and interact with the world in ways that were once thought impossible.

With regard to the biocompatibility concern, those recent studies all show that biocompatibility has made significant progress in material selection and tissue integration, and inflammatory responses have generally decreased. Flexible and nanomaterials can improve adaptability, and photovoltaic technology shows forward-looking potential, but long-term stability and electrode durability remain common challenges that require interdisciplinary collaboration to solve. Over years of implantation, encapsulation failures can occur and are a major concern. Common failure modes include moisture ingress through tiny defects, leading to gradual corrosion of electronics, and delamination of coatings due to mechanical stress (e.g., the separation of layers or cracking of a brittle coating). Such failures can drastically degrade electrode performance (including increased impedance or complete loss of function in affected channels) and pose patient safety risks if, for example, a hermetic breach allows bodily fluids to contact electronics or battery components. Therefore, hermetic sealing remains the gold standard for chronic implants. Modern retinal implant research often includes accelerated aging tests (e.g., soaking devices in 60 °C saline for months) to ensure encapsulations will last 5–10 years or more in vivo.

Last but not least, we highlighted that, even with perfect encapsulation, the body’s reaction to a foreign object plays a role. Chronic implants typically elicit a foreign body response—a thin layer of fibrotic scar tissue or gliosis can form around the device and electrodes. Over time, this can increase the distance between electrodes and neurons (raising stimulation thresholds) and isolate the implant electrically. Studies have shown that stiffer implants (or those with rougher surfaces) tend to induce more fibrosis and inflammation, whereas softer, more compliant materials cause a milder response. This is a key motivation for using flexible substrates and coatings. For retinal implants specifically, the presence of an array on or under the retina can lead to gliosis (Müller cell activation) at the interface. However, careful material choice (such as inert coatings and smooth surfaces) and drug-eluting strategies (like slow-release anti-inflammatory coatings) are being investigated to mitigate these tissue reactions.

## Figures and Tables

**Table 1:** Comparison of two major types of retinal implants.

Feature	Subretinal Implants	Epiretinal Implants
Target Cells	Bipolar cells	Ganglion cells
Electrode Placement	Beneath the retina (subretinal space)	On the retinal surface (vitreal side)
Power Source	Photovoltaic (light-powered) or wired (transocular cable)	External power (wireless induction or batteries in glasses unit)
Surgical Complexity	More invasive (retinal detachment and subretinal insertion)	Less invasive (device affixed to retinal surface)
Image Processing	Intrinsic retinal processing (implant directly uses light input)	External camera-based processing (real-time video feed)
Resolution Potential	Higher (finer pixel pitch possible, uses residual neural network)	Lower (limited by electrode count and size)
Adaptability	Limited adjustability post-implantation (fixed electronics)	Adjustable via external controls (camera and processor tunable)
Advantages	- Leverages residual retinal circuitry for more natural image processing. - No external camera needed (uses eye’s optics). - Typically higher spatial resolution than epiretinal implants (denser photodiode arrays).	- Easier surgical implantation and removal (no subretinal surgery). - Adjustable stimulation settings via external hardware/software. - Well-suited for advanced retinal degeneration where bipolar cells are non-functional (can stimulate ganglion cells directly).
Challenges	- Requires complex surgery (risk of retinal detachment during implantation). - Power constraints (for photovoltaic designs, ambient light may be insufficient; for wired designs, transscleral cables pose risks). - Variability in outcomes due to patient-specific subretinal anatomy (e.g., scar tissue can affect photodiode function).	- Depends on external camera (introduces some latency and artificial image processing). - Direct ganglion cell stimulation can produce unnatural visual percepts (patients often see discrete spots of light). - Lower spatial resolution relative to subretinal approaches (fewer, larger electrodes). Visual outcomes can be limited by eye movements misaligning the camera’s view with the implant.

**Table 2:** Comparison of selected retinal implant studies and systems [38,39,40,41,42,43,44,45,46,47].

Reference	Main Contribution	Advantages	Disadvantages	Placement Position of Electrode	Year
Chow et al., IEEE TNSRE [38]	Implanted silicon microphotodiode arrays in subretinal space (cat model).	Demonstrated biocompatibility and photodiode function in vivo.	Limited resolution; preclinical animal study (no human data).	Subretinal	2001
Zrenner et al., Vision Res. [39]	Explored subretinal microphotodiodes to replace lost photoreceptors.	Validated theoretical feasibility of subretinal implants.	Lacked functional validation in humans (concept study).	Subretinal	1999
Humayun et al., IOVS [40]	Morphometric analysis of postmortem RP patient retinas (no implant).	Provided anatomical insights for implant design (e.g., cell counts, spacing).	No implant tested—study of retinal structure only.	N/A (anatomy)	1999
Wang et al., J. Neural Eng. [41]	Photovoltaic retinal prosthesis: implant fabrication and performance (in vitro/in vivo tests).	Compact design; completely wireless operation.	Requires high-intensity illumination; tested in animals (preclinical).	Subretinal	2012
Boinagrov et al., J. Neural Eng. [42]	Compared stimulation selectivity of epi-, sub-, and intraretinal electrodes (computational and in vitro).	Detailed comparison of three placements; identified trade-offs in activating neural networks.	Theoretical/experimental study—not a clinical trial.	Epi-, Sub-, and Intra-	2014
Edwards et al., Ophthalmology [43]	Alpha AMS subretinal implant clinical trial in end-stage RP patients.	Showed partial restoration of visual perception in blind patients (light localization, object detection).	Limited field of view and modest resolution (approx. 1500 pixel implant).	Subretinal	2018
Chuang et al., Br. J. Ophthalmol. [44]	Systematic review of retinal implants (pre-2014 literature).	Summarized clinical and preclinical findings across studies.	No new experimental data (literature review).	Various	2014
Goetz & Palanker, Rep. Prog. Phys. [45]	Comprehensive technical review of electronic vision restoration approaches.	Provided broad overview with engineering insights.	Theoretical perspective, no new experiments (review).	Various	2016
Flores et al., J. Neural Eng. [46]	Optimized pillar electrode design for subretinal implants (in vitro study).	Enhanced proximity of electrodes to target neurons, improving stimulation efficacy.	Still in experimental phase (no human implantation yet).	Subretinal	2018
Wang et al., Nat. Commun. [47]	Demonstrated prosthetic vision matching natural acuity in rats (using a high-resolution subretinal optoelectronic implant).	Achieved near-natural resolution in an animal model (a significant milestone in acuity).	Preclinical results only; no human trials yet conducted.	Subretinal	2022

**Table 3:** Summary of representative recent electrode designs for retinal implants.

Advance (Year)	Electrode Design Details	Fabrication Techniques	Key Findings/Metrics
Soft Liquid–Metal Electrodes (2024)	Ultrathin (10 μm) flexible artificial retina with embedded eutectic Ga-In (EGaIn) liquid-metal microelectrodes (3D micropillars: 60 μm height, 20 μm diameter, Pt-nanocluster coated tips).	3D printing of micro-molds; pneumatic microfluidic filling of liquid metal; six-axis laser micromachining of electrode array.	High charge capacity (72.8 mC·cm^−2^) and low impedance (~210 kΩ at 1 kHz) achieved. In vitro: 82% cell viability; in vivo (rat) showed no inflammation at 5 weeks. Predicted visual acuity ~20/160 with high-density configuration.
Hexagonal 3D Pillar Array (2020)	Flexible PDMS-based array with 98 circular silicon pillar electrodes (150 μm diameter, 350 μm pitch, ~20 μm protrusion). Parylene-C insulation except at tips, platinum-coated.	Deep reactive ion etching (DRIE); chemical etching and elastic PDMS press; metal sputtering (Ti/Pt).	Impedance ~385 kΩ (1 kHz), CSC 2.83 mC·cm^−2^. Enhanced localized stimulation of retinal cells with reduced current spread. Demonstrated improved targeting of retinal neurons in vitro.
Monolithic LCP-Based Prosthesis (2013)	LCP microelectrode array with integrated circuitry. Thickened metal tracks; electrode sites laser-patterned.	High-pressure lamination; electroplating of gold conductors; laser ablation; monolithic encapsulation.	Achieved long-term stability in aging tests. Low moisture uptake. Improved mechanical reliability over polyimide or silicone-based arrays.
“Artificial Retina” Review (2019)	Survey of emerging concepts including organic polymer electrodes and 3D tissue-integrated electronics.	– (n/a, literature review) –	Reported novel approaches like conductive polymer electrodes and 3D structured electrodes. Emphasized micromachining for precise electrode patterning.
Intraretinal 3D Electrodes—NR600 (2024)	Intraretinal electrode array (~600 microelectrodes) placed within retina to stimulate neurons in multiple layers.	Custom 3D array fabrication; proprietary assembly; hermetic packaging.	Preliminary clinical results: Safe implantation in late-stage RP patients. Elicited finer phosphenes. Improved spatial resolution and contrast (early trial).

**Table 4:** Properties of selected electrode materials for retinal implants.

Material	Advantages	Disadvantages	Usage in Retinal Prostheses
Platinum (Pt)	Excellent biocompatibility; highly stable and corrosion-resistant; decades of use in implants.	Rigid (high modulus)—not flexible; relatively low surface area (unless textured); moderate charge injection limit.	Standard electrode material in most implants (e.g., Argus II uses Pt electrodes). Often used as base material due to reliability.
Iridium Oxide (IrOx)	Very high charge injection capacity (can deliver more charge without damage); forms capacitive, low-impedance interface when activated.	Brittle as a bulk material; requires activation/forming process; can dissolve or delaminate if overstressed over long term.	Used in some high-density arrays and penetrating electrodes to increase charge delivery (e.g., in experimental high-resolution electrode arrays). Common in lab prototypes needing low impedance.
Graphene	Ultra-thin and flexible; can be transparent; high electrical conductivity; large surface-to-volume ratio (potential for high charge storage).	New and relatively unproven long-term in vivo; challenges in making reliable electrical contacts; can be sensitive to defects (which may impact stability).	Investigated in recent research for flexible retinal electrodes and transparent electronics. Not yet in clinical devices, but promising for future microfabricated arrays that conform to retina.
Carbon Nanotubes (CNTs)	Extremely high surface area (greatly lowers impedance and stimulation threshold); good biocompatibility when purified; can form flexible, porous coatings on electrodes.	Difficult to pattern uniformly; risk of aggregation or fragmentation of nanotubes over time; long-term stability and integration still under study.	Used experimentally as coatings on metal electrodes to improve interface (e.g., lowering thresholds in animal tests). Showed that CNT-coated electrodes can integrate with retinal tissue over days, reducing threshold with time. Not yet in commercial devices.
Conductive Polymers (e.g., PEDOT:PSS)	Soft, tissue-like mechanical properties (minimizes foreign body reaction); can dramatically lower impedance; can be deposited on microelectrodes to improve charge transfer.	Prone to delamination or degradation in the body (swelling, cracking over months); limited operational lifetime if not encapsulated; some variants may leach by-products.	Being explored for ‘living electrodes’ that better integrate with neural tissue. PEDOT-coated electrodes in research have shown lower stimulation thresholds. Fully organic retinal implants (conducting polymer-based photodiodes) restored vision in rat models, though not yet in human trials.

**Table 5:** Comparative analysis of implantable encapsulation approaches (materials and techniques).

Encapsulation Type	Description	Advantages	Disadvantages
Thin Silicone Coating	Device components encased in medical-grade silicone (PDMS).	Very flexible; cushions device and dampens mechanical strain. Easy to apply.	Poor moisture/gas barrier (requires extra sealing layers); may allow slow fluid ingress → long-term failure if used alone.
Parylene-C Conformal Coating	Pinhole-free polymer film (µm-scale) vapor-deposited over device.	Excellent conformality, covering complex shapes; low permeability to moisture in short term; adds negligible thickness.	Brittle under repeated flexing; microcracks can form over time, compromising seal. Often used as secondary coating with other encapsulation.
Thin-Film Multilayer (Polymer + Oxide)	Stacks of alternating polymer and inorganic (oxide or nitride) layers. Example: polyimide plus silicon oxide layers.	Good barrier properties by complementary layers (polymer gives flexibility, oxide gives hermetic seal). Still relatively thin and flexible.	Fabrication can be complex; any pinhole in inorganic layer can let moisture in (hence, multiple layers needed). Long-term reliability under flex not fully proven.
Ceramic Package (e.g., Alumina or Glass)	Rigid case enclosing electronics, often brazed or bonded to metal feedthroughs.	Truly hermetic (virtually no moisture ingress); decades-long track record in implants (e.g., cochlear implants). Biocompatible and corrosion-proof.	Rigid and can be bulky relative to eye anatomy; requires precise assembly of feedthroughs; expensive fabrication.
Metal Case (Titanium or Stainless Steel)	Seam-welded metal can enclosing entire implant electronics, with electrode array fed through sealed connector.	Hermetic and extremely durable (titanium is biocompatible and an industry standard for hermetic implants). Excellent shielding of electronics.	Bulk and rigidity; not suitable for micro-scale or flexible retinal implants (would significantly limit conformability to eye). Also heavy.
Monolithic Encapsulation (LCP or Epoxy Housing)	Electronics embedded within a single-piece molded biocompatible material (like liquid crystal polymer) that acts as both circuit substrate and enclosure.	Fewer assembly steps (integrated solution); LCP in particular has very low moisture absorption and can be thermoformed to eye shape. Can be thin and conformable.	Achieving hermetic seals at contacts is challenging; some polymers still allow slight moisture ingress.

**Table 6:** Summary of implantable device packaging techniques.

Reference	Main Contribution	Advantages	Disadvantages	Application	Year
Cui et al.	Surface modification of neural electrodes with polymer/biomolecule blends	Improved biocompatibility and electrical interface	Potential degradation of coatings over time	Neural recording [95]	2001
Ding et al.	Flexible microelectrode arrays	Long-term stability, good neuronal interface	Complex microfabrication process	Chronic neuronal recording (cortex) [96]	2024
Koh et al.	Wearable microfluidic sweat sensors	Soft, flexible sensing	Low integration with rigid electronics (applicable more to wearables)	Wearable biosensing [97]	2016
Sreejith et al.	Biodegradable sensors for intracranial monitoring	Full biodegradability	Short operational lifespan	Brain pressure and temperature sensing…, etc. [98]	2023
Jeong et al.	Soft materials in neuroengineering	High compliance with tissue	Fabrication challenges	General neuroengineering applications [99]	2015
Fu et al.	Biodegradable optical fibers	Biocompatible light delivery	Limited transmission performance	Photomedicine [100]	2017
Hassler et al.	Parylene C characterization	Stable, low-permeability coating	Limited mechanical durability	Neural prosthesis encapsulation [85]	2011
Barrese et al.	Failure analysis of microelectrode arrays	Detailed mode identification	Silicon fragility	Intracortical electrodes [103]	2013
Fallegger et al.	Soft lithographic flexible interface	Good adaptability to cortex	Integration and alignment issues	Wireless BMIs [104]	2023
Hossain et al.	MEMS-enabled devices	Miniaturized systems	Packaging and power supply concerns	Implantable sensors [105]	2020
Ahn et al.	Comprehensive review of encapsulation	Covers multiple techniques	No novel experimental data	Reference for future designs [106]	2019
